# A Qualitative Study on the Development of Professionalism Among Japanese Dental Students

**DOI:** 10.7759/cureus.51762

**Published:** 2024-01-06

**Authors:** Takayuki Oto, Yuko Matsumoto, Yoichiro Iwashita, Reiko Yoshida, Norihiro Taguchi

**Affiliations:** 1 Department of General Dental Practices, Kagoshima University Hospital, Kagoshima-shi, JPN; 2 Department of Dental Education, Kagoshima University Graduate School of Medical and Dental Sciences, Kagoshima-shi, JPN

**Keywords:** medical professionalism, professionalism education, dental education, dental student, professionalism

## Abstract

Background: Professionalism is believed to vary depending on factors such as era and culture. Therefore, clarifying the meaning of professionalism in each country, region, and workplace is essential. However, how professionalism is cultivated among dental students in Japanese schools has yet to be fully elucidated. Therefore, this study examined whether professionalism among Japanese dental students changes by year. This research will contribute to effective professional education.

Participants and methods: The participants included six fourth-year dental students and nine fifth-year dental students. Semi-structured interviews were conducted from November 2018 to January 2019, and verbatim transcripts were created from the recorded data. Based on these verbatim transcripts, thematic analysis was utilized to examine and identify professionalism components for each academic year.

Results: Three themes based on 14 constituent concepts were obtained for fourth-year students. Three themes based on 20 constituent concepts were obtained for fifth-year students. Fourth-year students primarily focused on technical aspects. In contrast, fifth-year students placed greater emphasis on attitude and communication skills.

Conclusion: From fourth-year students, who primarily focus on classroom learning and practical training, to fifth-year students who gain clinical experience, the constituent elements of professionalism became more complex. However, this study did not examine other aspects of healthcare professionalism, such as interprofessional collaboration. A comprehensive education program tailored to the clinical setting is necessary for cultivating professionalism.

## Introduction

Professionalism has long been discussed as a critical theme in medical education [1]. For example, the Physicians' Charter [2], comprising three major principles, is a significant milestone in defining a doctor's professionalism. However, no universal definition exists within the medical profession [3]. Professionalism is not absolute, and it is said to vary with the era, country, and culture, subject to the changing values and other aspects prioritized in specific professional fields and by different nations [4]. In the field of dentistry, a 2020 report by the European Dental Education Association [5] presents various findings, including variations in professionalism based on the profession of dental healthcare providers. Therefore, clarifying professionalism in various professional domains and contexts is essential.

Professionalism education is of high importance in Japanese dental schools. It is listed at the top of the required competencies in the "Model Core Curriculum for Dental Education in Japan" [6], which comprises approximately 60% of the curriculum in Japanese dental schools.

However, research in Japanese dentistry is not significantly advanced. Our reports [7] indicate that the professionalism exhibited by Japanese dental trainees differs from that observed in other countries. For instance, when compared to the American Dental Education Association (ADEA) statement on professionalism [8], elements related to "Fairness" are notably absent in the values expressed by Japanese dental trainees. Conversely, pronounced emphasis is placed on the value of a "Career Path," reflecting a distinct set of professional priorities unique to this group. Although reports [9] on the professionalism of dental hygienists in Japan exist, those focusing on the developmental process of professionalism among students in dental schools are lacking. In particular, unique levels of professionalism exist at each university and in each academic year and are influenced by various factors. Studies on medical students have demonstrated that increasing experience affects communication and empathy [10, 11], and research on nurses has similarly suggested the influence of years of experience [12].

Furthermore, clinical clerkships significantly impact students [13, 14]. Clinical clerkships often begin in the fifth year in Japanese dental schools with a six-year curriculum. Significant changes may occur between the fourth and fifth years.

Understanding the development process of professionalism is crucial for implementing effective education. With a clear understanding of learner outcomes, reviewing educational content and guidelines is possible, and accurate assessment can be conducted.

Therefore, in this study, we aim to qualitatively analyze whether and how professionalism among dental students in Japan evolves throughout their education, specifically examining changes across each academic year. This exploration seeks to contribute to the broader understanding of medical professionalism, addressing the current gap in the literature regarding its developmental process in dental education.

Research question

How does the perception and manifestation of professionalism among dental students in Japan change across different academic years?

By addressing this question, our study aims to align the methods and outcomes of dental education with the actual developmental trajectory of professionalism, thus contributing significantly to educational theory and practical implementation in the field.

## Materials and methods

Research design

We conducted semi-structured interviews with students from the Faculty of Dentistry at Kagoshima University from November 2018 to January 2019. The interview records were analyzed using a qualitative data analysis method (thematic analysis) [15].

Participants and data collection

From November 2018 to January 2019, semi-structured interviews were conducted with students from the Faculty of Dentistry at Kagoshima University. All 46 fourth-year students and 43 fifth-year students were eligible for participation. Participants were randomly selected from those who agreed to take part in the study to ensure a diverse range of perspectives. In determining the number of participants, we aimed for approximately six participants, guided by the insights provided by Morse [16] on sample size in qualitative research. However, the final number of participants was decided upon confirming theoretical saturation - a state where no new themes were generated from the data - in accordance with the principles outlined by Flick [17]. This led to the final inclusion of six fourth-year students and nine fifth-year students. All interviews were conducted by one of the authors, "TO," ensuring consistency throughout the interview process. The average interview time was approximately 25 minutes per participant. Audio recordings were made during the interviews, from which verbatim transcripts were subsequently produced. As an example of the interview guide for semi-structured interviews, the guide includes questions such as: "Participants' backgrounds (e.g., motivations for enrolling in dental school)," "Qualities required of a dentist," "The ideal image of a dentist," "Good and poor instructors in lectures and practical training, and the reasons for their evaluation."

Educational curriculum for targeted students

Fourth-year students primarily study dentistry through lectures and practical training. They obtain some early exposure during their first year, but aside from that, they have no experience in clinical settings. In contrast, fifth-year students already have more than three months of clinical clerkship experience. This curriculum has remained unchanged since the initiation of our research in 2018. Consequently, the interview data’s value and derived findings remain pertinent and valid.

Data analysis

Data analysis was conducted via thematic analysis, a qualitative method, based on the verbatim transcripts we created. These processes were conducted in Japanese for each record of the interview subjects. The analysis was both retrospective and concurrent with the interview process, allowing for immediate reflection and adaptation as new data emerged. This approach ensured that by the time the final interviews were conducted, no new themes were generated, indicating that a theoretical saturation had been reached for each academic year. Therefore, concluding the interview process was deemed appropriate at this point. Finally, combining all the concepts, we developed integrated professionalism models for each year and their explanations. The analysis was conducted by one of the authors, “TO,” who had received training in qualitative analysis. Furthermore, all co-researchers collaborated to ensure the validity of the results.

Ethical considerations

This study was conducted with the approval of Kagoshima University's Ethics Committee for Epidemiological Research (No. 613, May 13, 2016). Additionally, careful attention was paid to ensure no psychological pressure was placed on the participants while obtaining informed consent and conducting interviews.

## Results

Upon generating theories from verbatim transcripts of the interviews, for fourth-year students, three themes based on 14 constituent concepts were identified, and for fifth-year students, three themes based on 20 constituent concepts. The themes and their descriptions are listed below.

Themes identified from interviews with the fourth-year students

Diverse Treatment Knowledge and Advanced Diagnostic Abilities

Being able to select various treatment methods and approaches appropriately based on the disease or symptoms and provide the most suitable treatment for the individual circumstances and needs of the patient.

Technical Excellence as a Dentist and Responsibility for the Overall Treatment

In dental treatment, manual skills are of utmost importance, and these technical skills directly impact the treatment outcomes and satisfaction of the patients. Dentists must constantly pursue cutting-edge techniques and methods, continually improving their skills. Additionally, dentists are responsible for the overall results of the treatment up to the point of healing. However, with high technical proficiency, one can compensate for deficiencies in other abilities.

Building and Respecting Relationships With People

By establishing a trusting relationship with patients, it becomes possible to provide long-term care. Understanding and empathizing with the patient's perspective and emotions allows for creating a deeper trust relationship. This bond forms the foundation for effective communication. Furthermore, by possessing fundamental values and attitudes as a human being beyond one's expertise as a dentist, building a genuinely trusting relationship with patients is possible.

Themes identified from interviews with the fifth-year students

Comprehensive Knowledge for Safe and Effective Treatment

Possessing fundamental medical knowledge about disease pathophysiology, diagnostic methods, and treatment modalities and the judgment to select the most appropriate treatment based on a patient's symptoms and test results contributes to optimizing healthcare quality. This expertise entails having the comprehensive knowledge necessary to provide safe healthcare to patients.

Excellence as a Dentist

Possessing the capability to excel beyond others in a specific area and apply this skill in treating patients.

Sincere Attitude and Excellent Communication Skills as a Dentist

Engaging in considerate actions such as offering reassuring words to alleviate patient anxiety, adopting a gentle demeanor without applying pressure, and clearly communicating one's treatment methods and opinions in a manner that patients can understand. Such communication facilitates the appropriate continuation of treatment and ultimately increases trust and satisfaction. Furthermore, by earnestly striving for the patient's welfare, a dentist can increase trust and satisfaction among patients and staff, showcasing their value as a provider of more effective healthcare services.

See also Tables 1-2 for the constituent concepts from both groups as well as examples of actual sentences.

**Table 1:** Concepts and sentence examples from fourth-year dental students

Fourth-year students	Theme	Constituent concepts	Examples of actual sentences
	Diverse treatment knowledge and advanced diagnostic abilities	Having a wide range of treatment options	I’d prefer a dentist with extensive knowledge who can offer a range of options, especially if I were the one receiving treatment.
		Knowledge to provide alternative choices, correct diagnostic skills	I think without knowledge, there's no progress. For example, there was an incident where a certain disease was overlooked, and it turned into a big issue. Well, I wouldn't call it an incident, but it was related to cancer and such. The outcome depends on whether you have the knowledge to question it or not.
	Technical excellence as a dentist and responsibility for the overall treatment	The area to focus on the most for the patient's benefit	I think knowledge is already a fundamental prerequisite, and when it comes to actually doing something with that knowledge, it's about skills.
		The importance of manual skills as a characteristic of dentistry	I believe that for dentists, performing actual procedures like drilling is more common than prescribing medications. It's these procedures that are crucial in producing key outcomes, making them extremely important.
		What is required for a dentist in terms of treatment	I do think that without the necessary skills, having other qualities might not make much of a difference.
		Having the technique to take responsibility for the entire healing process	Ultimately, skills are essential. After all, if you go to the dentist because of an issue in your mouth and it doesn’t get better, then the treatment is essentially pointless.
		Giving back everything one can to the patient	How can one fully utilize their abilities for the sake of the patients?
		Prioritizing problem-solving related to life	Therefore, even if a patient dislikes you, it's more important to have high abilities if it leads to their recovery and prolongs their life.
		Receiving favorable evaluations from anyone	An ideal dentist is not only a good dentist for the patients but also a good colleague among dentists; I think both are important.
		Leading to the final reputation	People tend to overlook a dentist’s bad attitude if they are skilled; competence seems to overshadow demeanor.
	Building and respecting relationships with people	Having an attitude that allows for longer treatment periods	In long-term dental care, it's not just about personal compatibility, but also about effective communication and having the right attitude with a reliable individual.
		The importance of empathy	I think it's really about understanding people's feelings, you know. Like many dentists say, don't do to others what you wouldn't want done to yourself, and what you can't do to your own parents or family, you shouldn't do to patients either.
		Human relationships rather than dentist-patient relationships	When it comes to treating patients, it may be a part of the service industry, but for me, the long-term relationship between individuals is also crucial. I believe a dentist needs to have a personable and approachable nature; if they don't, I wouldn't want them treating me.
		Fundamental human qualities before being a dentist	It’s more about behavior and character than just communication skills. Hmm... it's really about personality, I think. Without that, it's hard to even get started.

**Table 2:** Concepts and sentence examples from fifth-year dental students

Fifth-year students	Theme	Constituent concepts	Examples of actual sentences
	Comprehensive knowledge for safe and effective treatment	Knowledge to avoid harm to patients	Even if someone's not fully confident in their skills, having the knowledge to what shouldn't be done is crucial. With that understanding, they'll avoid making mistakes.
		Broad knowledge forming the basis of diagnosis and treatment	With a broader knowledge base and a well-informed perspective, you can examine patients. While technical skills may be necessary when examining patients, the most crucial aspect is foundational knowledge. I believe that knowledge is paramount.
	Excellence as a dentist	Having something exceptional	I feel like I must be a professional because I think I have something exceptional about myself, whether it's an outstanding personality or specialized skills.
	Sincere attitude and excellent communication skills as a dentist	Words to alleviate patient anxiety	Perhaps patients are thinking about what will be done to them in the future, maybe while having a towel placed on them or something. Since they don't really understand, it's probably good to explain not only what will be done now but also what will be done today or in the future. And maybe, if there's an incident, it's better to be able to explain it properly.
		Gentle attitude that does not pressure patients	Their words have a very gentle atmosphere, and they seem to lower themselves rather than looking down on the patients, so I think they are careful not to pressure the patients.
		The importance of altruistic attitudes	As a healthcare professional, I believe it's important not to lose the mindset of doing things for the sake of patients or others.
		Behaviors for the continuity of treatment	I think it's necessary to maintain a good attitude towards patients because if they perceive you as a bad dentist after just one visit, they probably won't come back.
		Responsibility and communication in explaining one's treatment	It's vital to take responsibility for the treatments performed and to have thorough discussions with patients, ensuring everything is explained properly.
		Genuine behavior as a true healthcare professional with heartfelt dedication	It's not necessarily wrong to use informal language with patients. There are various ways to express your attitude in Japanese, both in words and behavior. However, if there's no sincerity behind it, patients will feel it. Even if you speak politely, if patients feel belittled or condescended to, it creates a feeling of resistance. I've come across such situations now and then.
		Minimum skills expected of beginners	In the beginning, because my skills were lacking, I absolutely had to maintain a proper attitude, for starters. As I progress to higher stages, I believe that it's not just about attitude but also about continually adding knowledge and skills. So, I don't think I can definitively say which one is more important.
		Attitudes achievable specifically by that dentist	I got the impression that some people might only want to be treated by dentist N, who is adept at showing empathetic attitudes.
		Reliable skills that surpass attitude	Rather than saying the dentist had a bad attitude, it's more like they had a craftsman-like personality, were reserved, and didn't smile much, a quite remarkable character. Despite that, I still trusted them in terms of their skill.
		Attitudes towards people required to bring them for treatment	First and foremost, if the attitude toward people is not right, then even if they are undoubtedly skilled, it's probably... well, it feels unpleasant.
		Dental care that brings smiles to patients	Patients often talk among themselves about how great their dentist is, indicating a deep appreciation and liking for their dentist. Especially the dentist they are seeing appears to be very well-liked. The patients leave with a sense of "I'll be back, dentist," while smiling, and this ability to win the patients' trust is incredibly strong.
		Shaping the first impression of patients	Certainly, when patients first meet a dentist, the attitude is the first thing they notice, without a doubt.
		Representing the image of a dentist	They might appear ordinary, but in the examination room, they embody the role of a dental professional, reassuring you of their expertise.
		Trust gained from a frank attitude	Even though they speak casually to the elderly, you can feel a sense of trust due to their kindness.
		Valuing patients first	I think the most important thing for a patient, from their perspective, is the establishment of rapport. No matter how much knowledge and skill you have, if your attitude is poor, you won't gain their trust, and I believe it won't work out.
		Improvement even beyond treatment	It seems like there is such a deep connection. Just by maintaining that connection, not only in treatments but also in other aspects, I believe that the treatment will certainly go well.
		Earning trust from patients and staff	Is it possible to consistently consider the other person and work with their needs in mind? If you are working within an organization, I believe that the quality of a job can change significantly based on whether you can truly think about your colleagues, staff, and peers in the same profession.

## Discussion

This study identified the constituent elements of professionalism for fourth- and fifth-year dental students based on their interview transcripts. Our findings indicate that fourth-year students placed a somewhat greater emphasis on technical excellence. It was more prominent than other constituent concepts. A belief was expressed that having technical skills could compensate for deficiencies in other areas. In the fourth year, the curriculum primarily focuses on classroom lectures and practical training, inevitably resulting in a more pronounced retention of knowledge and technical skills. Particularly in Japan, dental education has traditionally followed a "completion education" model, which aims to produce dentists capable of independent practice with specific acquired skills by graduation [18, 19]. As a result, many educators emphasize the acquisition of technical skills. Such factors may influence students who believe "technical skills are more important." "Excellence" is one of the elements in Stern's model [20]. Since neglecting other aspects is inadvisable, emphasizing the importance of aspects beyond technical skills is essential.

For fifth-year students, a prominent feature was the high number of constituent concepts focusing on attitudes, such as sincerity and communication skills. The clinical clerkship experience in the fifth year likely contributed significantly to a shift in students' attitudes, as observed in their direct interactions with faculty and patients. Notably, an enhanced recognition of the importance of "being able to initiate treatment" and "sustaining treatment" emerged. The prevailing view is that regardless of the extent of one's exceptional skills or knowledge, they are of no significance if there is no situation to apply them. Stern's model [20] comprehensively includes aspects such as communication skills, humaneness, accountability, and altruism, constituting a broader spectrum of constituent concepts compared to those understood by fourth-year students.

Our results show that fifth-year students were required to achieve theoretical saturation to a greater extent. A more significant number of theoretical descriptions was obtained, suggesting that professionalism becomes increasingly complex as they progress through their academic years and accumulate experience. Professional identity refers to their values, beliefs, and attitudes [21], of which professionalism is at the core. Professional identity formation is influenced by factors including “exposure to the business of medicine” and “exposure to physicians in clinical practice” [22]. Therefore, the impact of clinical clerkships, particularly between the fourth and fifth years of study, may play a significant role in advancing students' growth toward becoming more akin to dentists.

Moreover, compared to the professionalism model by Swick et al. [23], our study did not reveal elements related to societal engagement, academic activities, or lifelong learning. This difference might be due to the university hospital-based clinical training, which focused more on one-to-one relationships between dentist and patient. Additionally, a study on the professionalism of medical students in Taiwan, a region with some similarities to Japan, identified three key elements as most important: accountability to patients, respect for patients and their families, and integrity and prudence [24]. In our model, the concept of accountability is encompassed within "excellent communication skills," while "respect for patients and their families" as well as "integrity and prudence" are included in elements such as "respecting relationships with people" and "sincere attitude and excellent communication skills." This similarity suggests that there may be common underlying values in professionalism within the Asian context.

Extending these comparisons to other healthcare professions, our analysis further explores dental student professionalism. In our analysis, dental student professionalism is characterized by a strong emphasis on technical skills and patient relationships, showing interesting parallels and contrasts with other healthcare professions. Compared to the General Medical Council's model for physicians [25], both models value patient-centered care, effective communication, and trust. However, the physicians' model places more emphasis on broader patient safety and public health aspects, while dental students, especially in their fourth year, focus more on technical proficiency and dentistry-specific skills. When compared to the International Council of Nurses' model for Nurses [26], we noticed key differences. The nurses' model emphasizes holistic, person-centered care, human rights, and social justice. In contrast, dental students focus more on developing technical skills and patient care within a more specific clinical context. Though both prioritize patient welfare and ethical practice, nurses engage more in broader health advocacy and community involvement, offering a more comprehensive healthcare view. Additionally, when compared with the American Physical Therapy Association's professionalism model for physical therapists [27], dental students share values of excellence and accountability, focusing on technical proficiency and ethical practice. However, physical therapists emphasize broader societal responsibilities and altruism, reflecting a commitment to overall community health and well-being. These comparisons underscore the unique yet complementary roles each profession plays in the healthcare system, emphasizing the multifaceted nature of medical professionalism.

In our study, it became clear that professionalism among dental students is composed of elements including technical excellence, diverse treatment knowledge, sincere attitude, and excellent communication skills. Additionally, other critical elements from various models of professionalism, such as accountability and altruism [19], along with interprofessional collaboration, self-improvement, and lifelong learning from the Japanese dental hygienist model [9], are indispensable for healthcare professionals. Therefore, it is crucial to design an educational curriculum that comprehensively addresses these aspects of professionalism. Implementing early practical training and community dental clerkships can be beneficial, allowing students to gain firsthand experience with the realities of the field and dentist actions from an early stage. Incorporating these identified elements of professionalism into the curriculum should bring novel educational benefits.

Limitations

As this study is based on a limited sample from a single university, its findings may not extend to all dental students in Japan. However, the findings could apply to students in similar circumstances. The study focused only on the distinctive periods of the fourth- and fifth-year students, highlighting the necessity for future research to clarify the progression from the first to the sixth year. Due to COVID-19’s impact, we were unable to conduct further interviews. However, we plan to expand the study to include different academic years and universities to enhance the accuracy of the research.

## Conclusions

This study yielded distinct professionalism models for fourth- and fifth-year dental students in Japan. Notably, fourth-year students without clinical clerkship experience tended to prioritize technical skills. Conversely, fifth-year students with clinical clerkship experience showed a tendency to emphasize attitudes such as communication skills. This difference underscores the importance of integrating attitude development into the educational curriculum from the early stages. We propose that these findings offer new insights for developing improved curricula that cultivate professionalism.
